# Characterisation of the Maternal Response to Chronic Phase Shifts during Gestation in the Rat: Implications for Fetal Metabolic Programming

**DOI:** 10.1371/journal.pone.0053800

**Published:** 2013-01-14

**Authors:** Tamara J. Varcoe, Michael J. Boden, Athena Voultsios, Mark D. Salkeld, Leewen Rattanatray, David J. Kennaway

**Affiliations:** Robinson Institute, University of Adelaide, Adelaide, South Australia, Australia; Nagoya University, Japan

## Abstract

Disrupting maternal circadian rhythms through exposure to chronic phase shifts of the photoperiod has lifelong consequences for the metabolic homeostasis of the fetus, such that offspring develop increased adiposity, hyperinsulinaemia and poor glucose and insulin tolerance. In an attempt to determine the mechanisms by which these poor metabolic outcomes arise, we investigated the impact of chronic phase shifts (CPS) on maternal and fetal hormonal, metabolic and circadian rhythms. We assessed weight gain and food consumption of dams exposed to either CPS or control lighting conditions throughout gestation. At day 20, dams were assessed for plasma hormone and metabolite concentrations and glucose and insulin tolerance. Additionally, the expression of a range of circadian and metabolic genes was assessed in maternal, placental and fetal tissue. Control and CPS dams consumed the same amount of food, yet CPS dams gained 70% less weight during the first week of gestation. At day 20, CPS dams had reduced retroperitoneal fat pad weight (−15%), and time-of-day dependent decreases in liver weight, whereas fetal and placental weight was not affected. Melatonin secretion was not altered, yet the timing of corticosterone, leptin, glucose, insulin, free fatty acids, triglycerides and cholesterol concentrations were profoundly disrupted. The expression of gluconeogenic and circadian clock genes in maternal and fetal liver became either arrhythmic or were in antiphase to the controls. These results demonstrate that disruptions of the photoperiod can severely disrupt normal circadian profiles of plasma hormones and metabolites, as well as gene expression in maternal and fetal tissues. Disruptions in the timing of food consumption and the downstream metabolic processes required to utilise that food, may lead to reduced efficiency of growth such that maternal weight gain is reduced during early embryonic development. It is these perturbations that may contribute to the programming of poor metabolic homeostasis in the offspring.

## Introduction

It has become increasingly clear that early environmental influences can have long lasting impact on the normal development and physiology of the fetus. Factors such as poor maternal nutrition, prenatal stress and exposure to medicinal and social drugs can all have negative health consequences for the offspring that persist into adulthood [1]–[3]. This led us to consider whether exposure to abnormal lighting conditions during pregnancy, as occurs during maternal shift work, can also have life-long impacts on the developing fetus.

Due to the wide variations in the types and durations of working schedules conducted in the community, it can be difficult to experimentally expose either human subjects or animal models to ‘shift work’. Nevertheless, shift work is characterised by forced disruptions in the timing of activity, sleep and light exposure, leading to disordered endocrine, metabolic and behavioural rhythms. While it is known from epidemiological and experimental studies that these changes can increase the risk to the individual of developing a myriad of chronic health disorders, it also raises the important question of whether exposure to shift work during pregnancy can also affect the developing fetus.

To address this question, we recently exposed pregnant rats to a chronic phase shift (CPS) protocol whereby the photoperiod was reversed twice every week throughout gestation and for 1 week after birth. We demonstrated that *in utero* and early postnatal exposure to this protocol negatively affected metabolic homeostasis, with offspring displaying age and gender dependent hyperleptinaemia, hyperinsulinaemia, poor glucose tolerance, insulin resistance and increased adiposity [4]. These changes occurred despite there being no effect of the protocol on birth weight or early postnatal growth. Manipulating the photoperiod in this way is likely to disrupt behavioural, endocrine and metabolite rhythms, as well as deregulate the expression of clock genes. How these disruptions translate into altered metabolic programming in the offspring, however, is unknown.

In this study we investigated the impact of chronic phase shifts on maternal and fetal hormonal, metabolic and circadian rhythms. Pregnant rat dams were exposed to either a control photoperiod or chronic phase shifts of the photoperiod, and maternal weight gain, food consumption, the circulating hormone and metabolite concentrations, and glucose and insulin tolerance were assessed. We also assessed the impact of this protocol on 24 hour profiles of clock and metabolic gene expression in the maternal and fetal liver.

## Methods

### Animals

All experiments were approved by the University of Adelaide Animal Ethics Committee and were conducted in accordance with the Australian Code of Practice for the Care and Use of Animals for Scientific Purposes. Albino wistar female rats aged 7–9 weeks were housed with males (2∶1) until pregnancy was confirmed by vaginal smears for sperm. Upon detection of mating, females were housed individually and maintained on either control lighting conditions (12 light:12 dark, lights on at 0800 h) or exposed to repeated phase shifts throughout gestation (presence of sperm, day 1). The chronic phase shift (CPS) protocol involved manipulating the lighting schedule so that every 3–4 days the photoperiod was reversed (Figure S1). The photoperiod reversal occurred on the night of day 1 of gestation, so that rather than going off at 2000 h as expected, the lights remained on until 0800 h of day 2. Rats were weighed throughout gestation, and food consumption was calculated by weighing the food in the hopper at the end of each week (Control n = 9, CPS n = 12). An additional experiment was conducted to analyse the timing of food consumption. Upon the presence of sperm, individually housed dams were exposed to either the control or CPS protocol, with food consumption recorded every minute (LabMaster Control System, TSE Systems, Bad Homburg, Germany) until parturition (Control n = 4, CPS n = 5).

### 24 Hour Analysis of Hormones, Metabolites and Liver Clock Gene Expression

At day 19 to 20 of gestation, a separate cohort of pregnant dams were killed by decapitation at 2000, 2400, 0400, 0800, 1200 and 1600 h (n = 4–6 per treatment, per time point, total of 64 dams). An additional group of 10 non-pregnant control females of comparable age were killed at 0800 h (n = 10). Major organs including brain, heart, liver, kidney, gastrocnemius muscle, epigonadal fat, retroperitoneal fat, spleen, pancreas, stomach and adrenals were rapidly dissected, weighed and stored in RNAlater® (Life Technologies, Carlsbad, CA) for gene expression analyses. Trunk blood was collected into lithium-heparin tubes. The number of pups was determined, and the second pup and placenta from each horn were dissected and weighed (L2 and R2). The placentae were separated into the labyrinth and junctional zones, and separately stored in RNAlater®, as was fetal liver. Plasma concentrations of glucose, cholesterol, free fatty acids and triglycerides were assayed by colorimetric enzymatic analysis on a Hitachi automated centrifugal analyzer with the use of kits from Roche Diagnostics (Castle Hill, Australia). Melatonin, corticosterone, insulin and leptin were assayed by RIA using kits obtained from Buhlmann (Schonenbuch, Switzerland), MP Biomedicals (Orangeburg, NY) and Linco Research (St. Charles, MO) respectively.

### Glucose and Insulin Tolerance Tests

At day 20 of gestation, separate cohorts of pregnant rats were subjected to intraperitoneal glucose tolerance (IPGTT) and insulin tolerance tests (IPITT). These occurred 26–28 hours after resumption of a normal photoperiod, and 2–3 hours after lights on. For the IPGTT, overnight fasted animals were injected intraperitoneally with glucose (2 g/kg body wt; n = 7 per treatment group). Blood was obtained from the tail vein before and 15, 30, 60, 90 and 120 min after glucose administration. Glucose was analysed by the dehydrogenase method (HemoCue, Angelholm, Sweden), with additional blood collected into lithium-heparin microvettes at all time points for subsequent insulin assay. For the IPITT, food was withdrawn at the beginning of the test and the rats were injected intraperitoneally with insulin (0.75 IU/kg body wt; Actrapid, Novo Nordisk; n = 7 per treatment group) 2–3 hours after lights on. Blood (5 µl) was obtained from the tail vein before and 5, 10, 15, 30, 60, 90 and 120 minutes after insulin administration for glucose determination.

### Gene Expression Analysis

RNA was isolated from 100 mg of maternal liver, 100 mg of placental labyrinth or 50 mg of fetal liver using TriReagent (Sigma, St Louis, MO) according to the manufacturer’s protocol. Only 1 fetus and placenta from each dam was processed for gene expression analysis, corresponding to position L2. Residual contaminating DNA from all samples was removed using Ambion DNA*free*™ kits (Applied Biosystems, Foster City, CA, USA). First strand cDNA was generated from 2 µg of RNA using Invitrogen Superscipt III reverse transcription kits (Invitrogen Corporation, Carlsbad, CA). Amplification of cDNA was performed on a GeneAmp 7500 Sequence Detection System (Applied Biosystems, Foster City, CA) in duplicate, using primers designed and optimised in our laboratory (Table S1). The expression of each gene within each sample was normalised against *β-actin*, and expressed relative to a calibrator sample with the use of the formula 2^−(ΔΔCt)^ as described previously [5].

### Statistics

Statistical analyses were conducted using SPSS v.18 or GraphPad Prism 5. Maternal weight gain and food consumption from the control and CPS groups was fit to a polynomial quadratic equation (Y = B0+B1*X+B2*X∧2). Food consumption data from the LabMaster system was transferred to an Excel document where the amount of food consumed for each rat in each hour throughout gestation was calculated. Litter size was analysed using non-parametric Mann-Whitney tests. Fetal, placental and fetal:placental ratio data was analysed using two-factor ANOVA. Hormonal, metabolite and gene expression data was analysed using two-factor ANOVA (time and treatment as factors) with Bonferroni post hoc analyses to compare individual time points. Area under/above the curve in the glucose and insulin tolerance tests was analysed using t-tests. The probability value used to identify statistical significance was P<0.05. 24 hour data was also fit to a sine curve where the frequency was 24 hours, with the amplitude, peak and trough values determined using Circwave (http://hutlab.nl), with a probability of P<0.05 considered rhythmic.

## Results

### Growth Trajectories and Food Consumption

**Figure 1:**
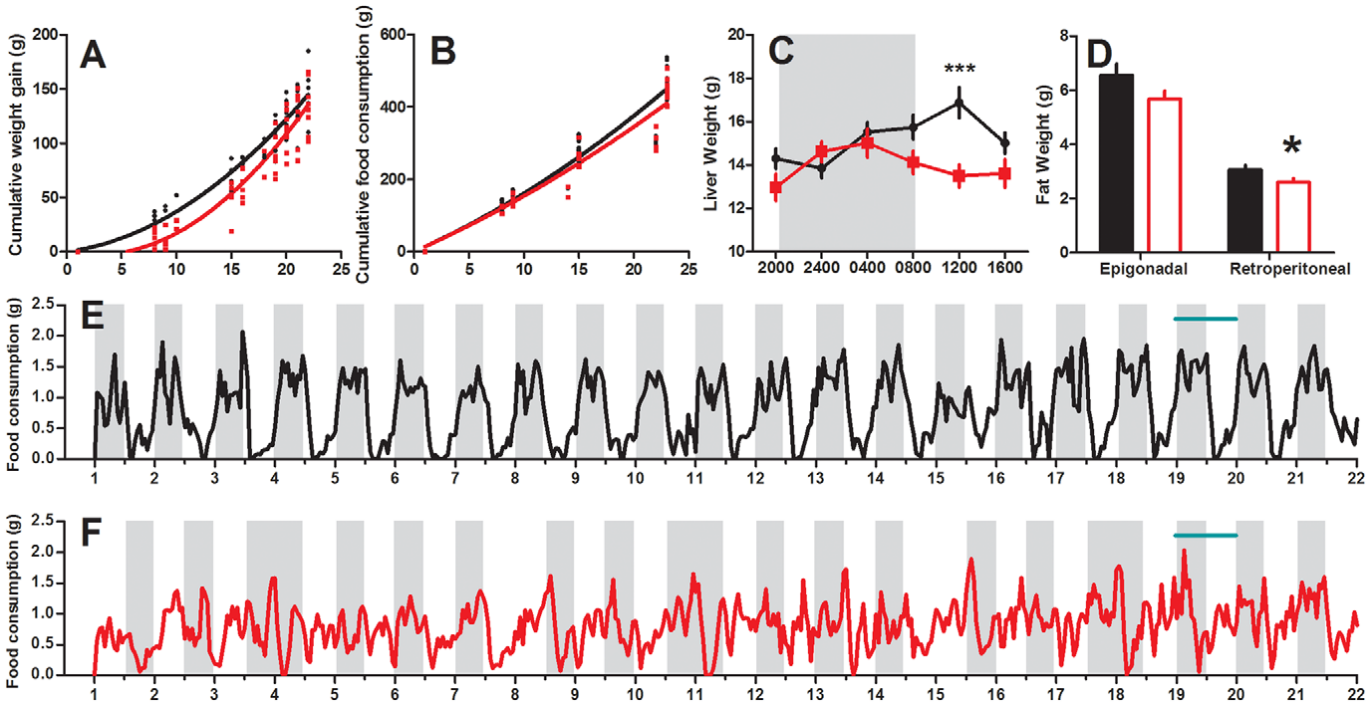
Maternal weight gain, food consumption, adiposity and liver weight in response to chronic phase shifts. Weight gain (a), total food consumption (b) day 20 liver (c) and epigonadal/retroperitoneal fat weight (d) of rats exposed to chronic phase shifts (CPS, red) or normal lighting conditions (control, black) throughout gestation, *P<0.05, ***P<0.001. The timing of food consumption throughout gestation in control (e) and CPS (f) dams. Horizontal green bar represents the period of tissue collection, and shading the timing of darkness in each group.

The weight gain of control and CPS dams was fitted to the polynomial quadratic equation (r = 0.92 and 0.91 respectively), with comparisons between groups revealing significant differences in B1 (P<0.001) and B2 (P<0.01, Figure 1a), suggesting differences in weight gain over gestation. During the first week, the control dams gained on average 25 grams, compared to only 8 grams in the CPS dams. Weight gain was parallel in the control and CPS dams during the second week (48 and 45 g respectively), however in the third week, the CPS dams gained 12 grams more than the controls, so that over the whole gestational period they had gained only 9 grams (6%) less. At day 19 of gestation, when animals were weighed for the 24 hour data collection, CPS dams weighed 5% less than the controls (Control 380±5 g, CPS 360±6 g, P<0.05). Cumulative food consumption for both groups also fitted a polynomial quadratic equation (r = 0.98), although there was no significant difference in either B1 or B2 (P>0.05), or total food consumed (Control 479±13 g, CPS 450±10 g, P>0.05), suggesting no differences in total food consumption between the control and CPS dams at any stage of gestation (Figure 1b). Analyses of the timing of food consumption reveal that control animals consolidate their feeding to night, with 80% of their food being consumed between 2000 h and 0800 h. In contrast, the CPS dams graze consistently over 24 hours, on average eating only 57% of their food between 2000 h and 0800 h. Even 3 days after resumption of a normal photoperiod, the CPS dams still only eat 65% of their food at night. Furthermore, this data confirms that total food consumption at any stage of gestation was not affected by treatment (Table S2, p>0.05). Body composition analyses performed at day 20 of gestation revealed liver and retroperitoneal fat was significantly reduced in the CPS dams (Figure 1c–d). For the liver, there was a significant interaction effect of time and treatment, with *post hoc* Bonferroni analyses revealing a significant decrease at 1200 h (P<0.05). There was no effect of treatment on the weight of any of the other tissues analysed (Table 1).

**Table 1:** Body composition analyses of pregnant and non-pregnant dams.

	Control- not pregnant	Control- pregnant	CPS- pregnant
Body weight	244.8±9.50	380.4±5.49*	360.4±6.14*
Pancreas	0.528±0.024	0.716±0.023*	0.698±0.024*
Spleen	0.574±0.031	0.764±0.017*	0.73±0.018*
Epigonadal Fat	4.971±0.624	6.549±0.424	5.678±0.305
Retroperitoneal Fat	2.077±0.214	3.072±0.461*	2.604±0.159#
Adrenals	0.061±0.004	0.077±0.003*	0.07±0.002
Kidneys	1.832±0.054	2.108±0.027*	2.102±0.038*
Heart	0.817±0.025	0.999±0.016*	0.952±0.019*
Liver	10.237±0.336	15.22±0.274*	13.939±0.253* #
Gastrocnemius muscle	2.864±0.074	3.283±0.062*	3.223±0.07*
Brain	1.886±0.034	1.958±0.019	1.986±0.02

* significantly different to non-pregnant controls, P<0.05.

# significantly different to pregnant controls, P<0.05.

Pregnant dams were killed at 4 hour intervals over 24 hours (n = 32 per group), whereas non-pregnant animals were killed at 0800 h (n = 10).

### Fetal and Placental Growth

**Figure 2:**
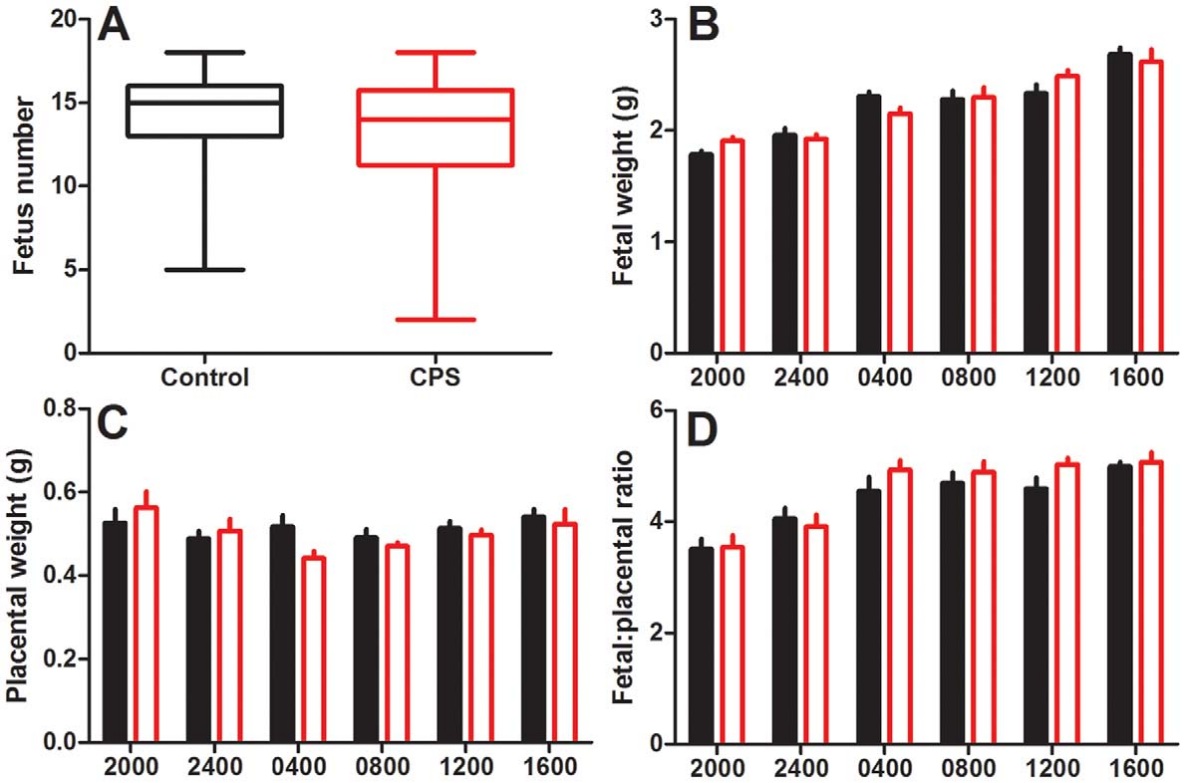
Litter size, fetal weight and placental weight in response to chronic phase shifts. Litter size (a), fetal weight (b), placental weight (c) and the fetal:placental ratio (d) of rats exposed to chronic phase shifts (CPS, red) or normal lighting conditions (control, black) throughout gestation. Fetus number is expressed using a box plot with the median, minimum and maximum values (whiskers) and the inter-quartile range (n = 32 per treatment). Fetal and placental weight and ratio are 2 representative fetuses/placenta from each litter, corresponding to position L2 and R2, mean ± SEM, n = 8–12 per group.

There was no effect of treatment on litter size (Figure 2a). Analysis of fetal weight over the period of day 19 to 20 revealed significant weight gain, with fetuses gaining 890 mg (50%) from 2000 h on day 19 to 1600 h on day 20 (Figure 2b). Placental weight however, changed little over this time period (Figure 2c). Calculations of fetal:placental ratio revealed an increase from 3.5 to 5 over the sampling times (Figure 2d). These results highlight the rapid fetal growth and increases in the fetal:placental ratio that occur during late gestation. There was, however, no effect of CPS for either fetal weight, placental weight, or the fetal: placental ratio, consistent with no change in birth weight being observed following this protocol [4].

### Maternal Hormone and Metabolite Concentrations

**Figure 3:**
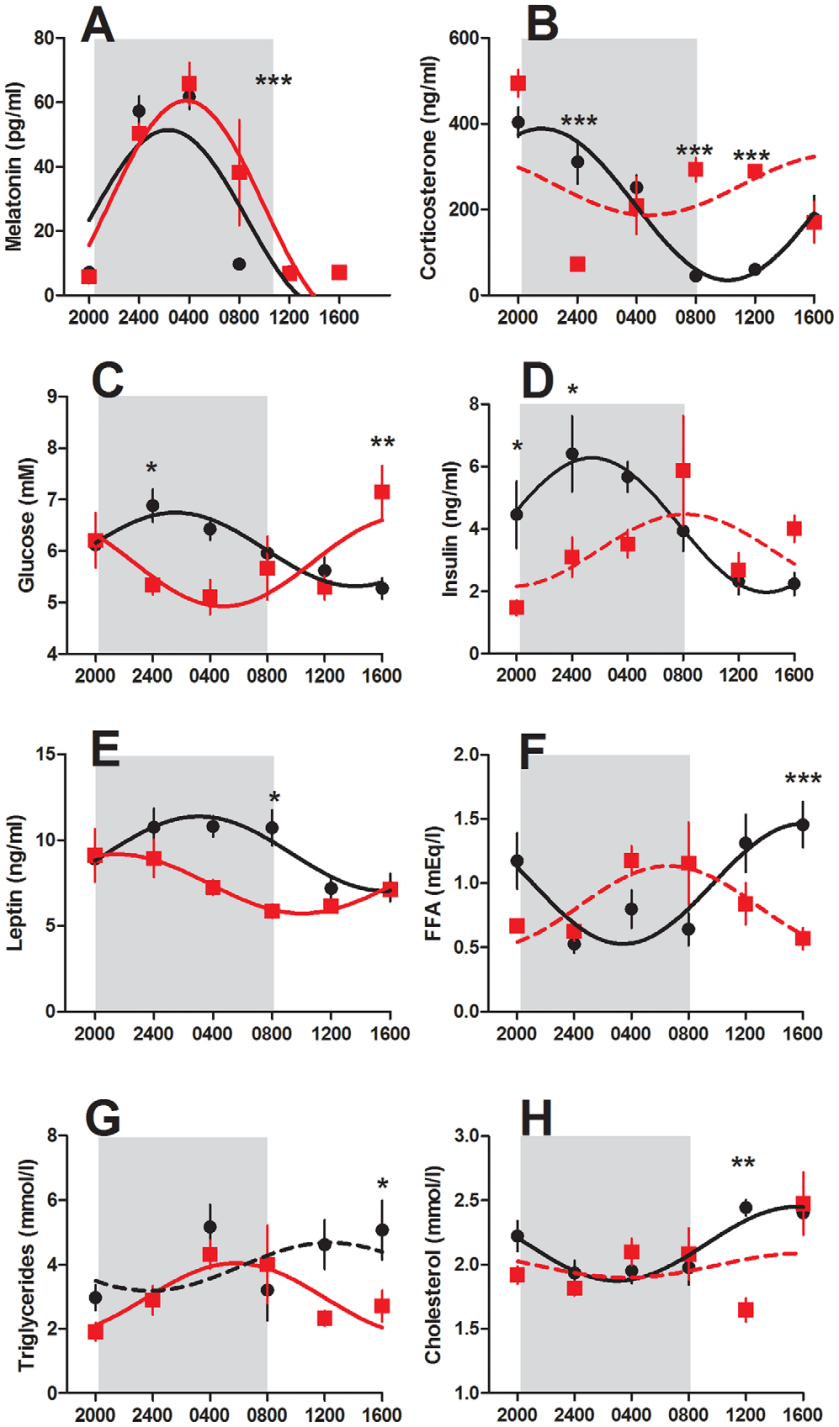
Circadian profiles of maternal hormone and metabolite concentrations. Melatonin (a), corticosterone (b), glucose (c), insulin (d), leptin (e), free fatty acids (f), triglycerides (g) and cholesterol (h) of rats exposed to chronic phase shifts (CPS, red square) or normal lighting conditions (control, black circle) throughout gestation. Shading represents darkness in both groups. Data has been fit to sine curve with the period constrained to 1, with solid lines representing a significant fit and dashed lines a non-significant fit. Data is mean ± SEM, n = 4–6, *P<0.05, **P<0.01, ***P<0.001.

As expected, plasma melatonin concentrations were elevated at night and low during the day in the control animals, with cosinor analysis revealing a significant fit to the sine curve (P<0.001), and therefore considered to be rhythmic (Figure 3a). The profile of melatonin secretion was similar in the control and CPS dams, with high concentrations of this hormone occurring during darkness, and low levels in the light. Two factor ANOVA confirms that there was no effect of treatment on the expression of this hormone, however, there was a treatment x time interaction effect (P<0.01), due solely to the difference between the two groups at 0800 h, corresponding to the time of lights on.

By contrast, plasma corticosterone levels were strikingly different between the two groups. In the control animals, corticosterone secretion was rhythmic (P<0.001, Figure 3b), with peak concentrations at the start of the dark phase (2236 h), gradually decreasing until the time of lights on (1036 h). Levels remained low for the next 4 hours, before gradually increasing towards the end of the light phase. The CPS dams however, did not show rhythmic secretion of this hormone (P>0.05), with high concentrations being observed at 2000 h, which rapidly declined to 80 ng/ml at 2400 h, before gradually increasing over the remaining sampling times. These differences were reflected in a significant treatment effect (P<0.05), with *post hoc* Bonferroni analyses revealing lower concentrations of this hormone at 2400 h (P<0.001) and elevated concentrations at 0800 h and 1200 h (P<0.001) in the CPS dams.

Analysis of the plasma metabolites in the control dams revealed rhythmic concentrations of glucose (P<0.05, Figure 3), insulin (P<0.001), leptin (P<0.001), cholesterol (P<0.01) and free fatty acids (P<0.01), with only the levels of triglycerides not fitting a sine curve (P>0.05). Both glucose and insulin were elevated during darkness, or the expected time of food consumption, while cholesterol and free fatty acids were elevated during the light period, or time of expected sleep.

Despite resumption of a normal photoperiod prior to sample collection, the concentration of these metabolites in the CPS treated dams was profoundly different. Glucose levels changed rhythmically over the 24 hours (P<0.05) with low levels during darkness and subsequently increasing during the following light period. This was reflected in a significant treatment by time interaction (P<0.05) whereby CPS dams had reduced levels at 2400 h (P<0.05), and higher levels at 1600 h (P<0.01). Similarly, insulin concentrations varied differently with time (P<0.01) such that CPS dams had reduced levels at 2000 h and 2400 h (P<0.05). Plasma leptin concentrations were significantly affected by treatment (P<0.001), being reduced during the late darkness period in the CPS dams (P<0.05), whereas free fatty acid concentrations varied differently with time such that CPS dams had reduced concentrations at 1600 h (P<0.001). Both cholesterol and triglyceride concentrations were affected by treatment (P<0.05), with *post hoc* Bonferroni analyses revealing differences at 1200 h and 1600 h respectively.

### Glucose and Insulin Tolerance Tests

**Figure 4:**
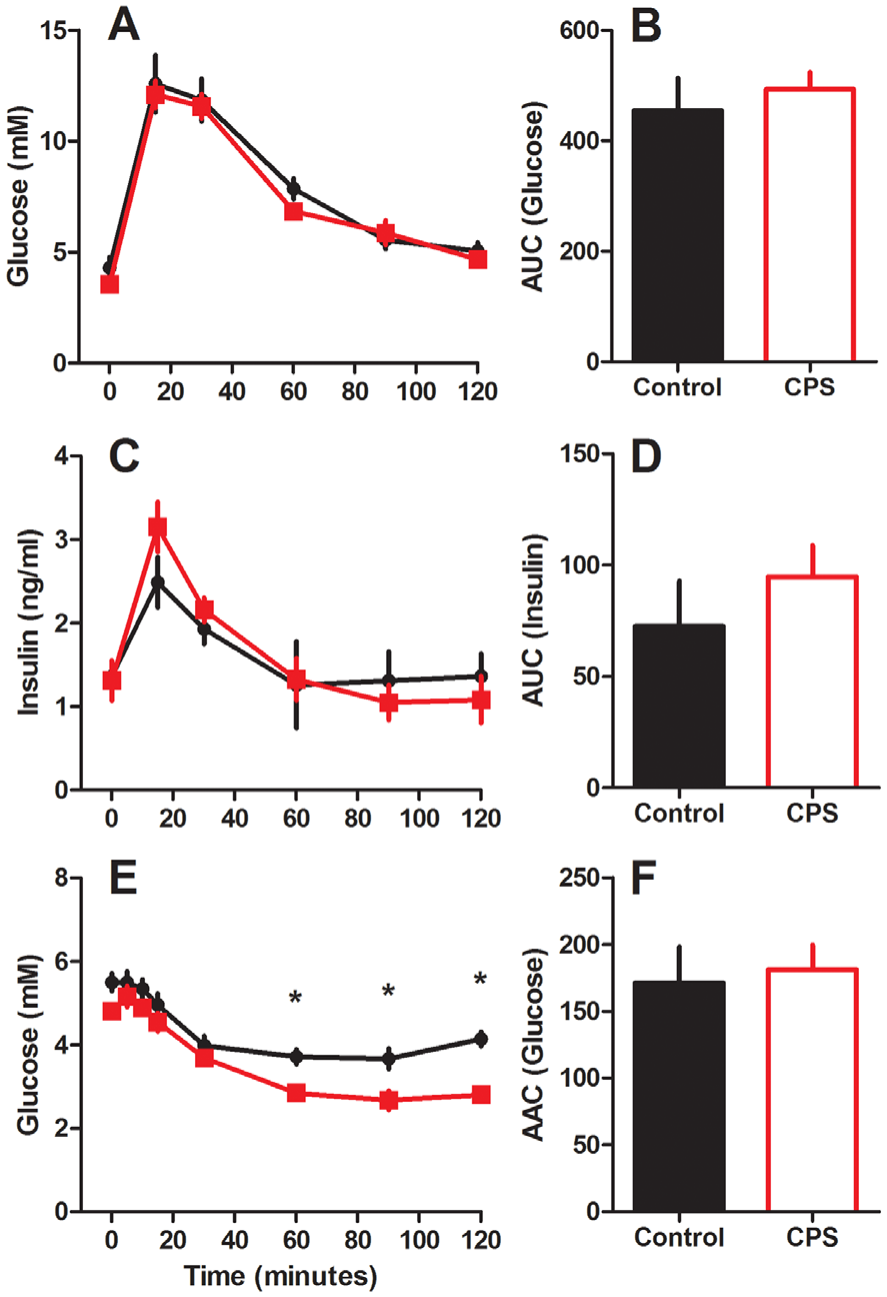
Glucose and insulin tolerance during gestation. Blood glucose (a) and plasma insulin (c) following 2 g/kg intraperitoneal injection of glucose with corresponding area under the curve (b and d), or blood glucose (e) and corresponding area above the curve (f) following 0.75 IU/kg intraperitoneal injection of insulin to pregnant dams exposed to chronic phase shifts (CPS, red square) or normal lighting conditions (control, black circle) throughout gestation. Data is mean ± SEM, n = 7 per treatment group.

The glucose response to IPGTT did not differ significantly between the control and CPS dams as reflected in the area under the curve (AUC, P>0.05, Figure 4). Likewise, the insulin response to glucose administration was similar in both groups (P>0.05). The profile of blood glucose response to insulin administration however differed, with repeated measures ANOVA revealing a significant difference in blood glucose concentrations at 60, 90 and 120 minutes post insulin administration (P<0.05). However, the change in glucose from baseline to 30 minutes post treatment did not differ between the two groups (Control 1.41±0.3, CPS 1.11±0.18, P>0.05), and when area above the curve (AAC) was calculated relative to baseline, there was no effect of treatment (P>0.05), suggesting that while baseline glucose are reduced in the CPS dams (−0.7 mM), the sensitivity to insulin was not altered.

### Maternal Liver Clock Gene Expression

**Figure 5:**
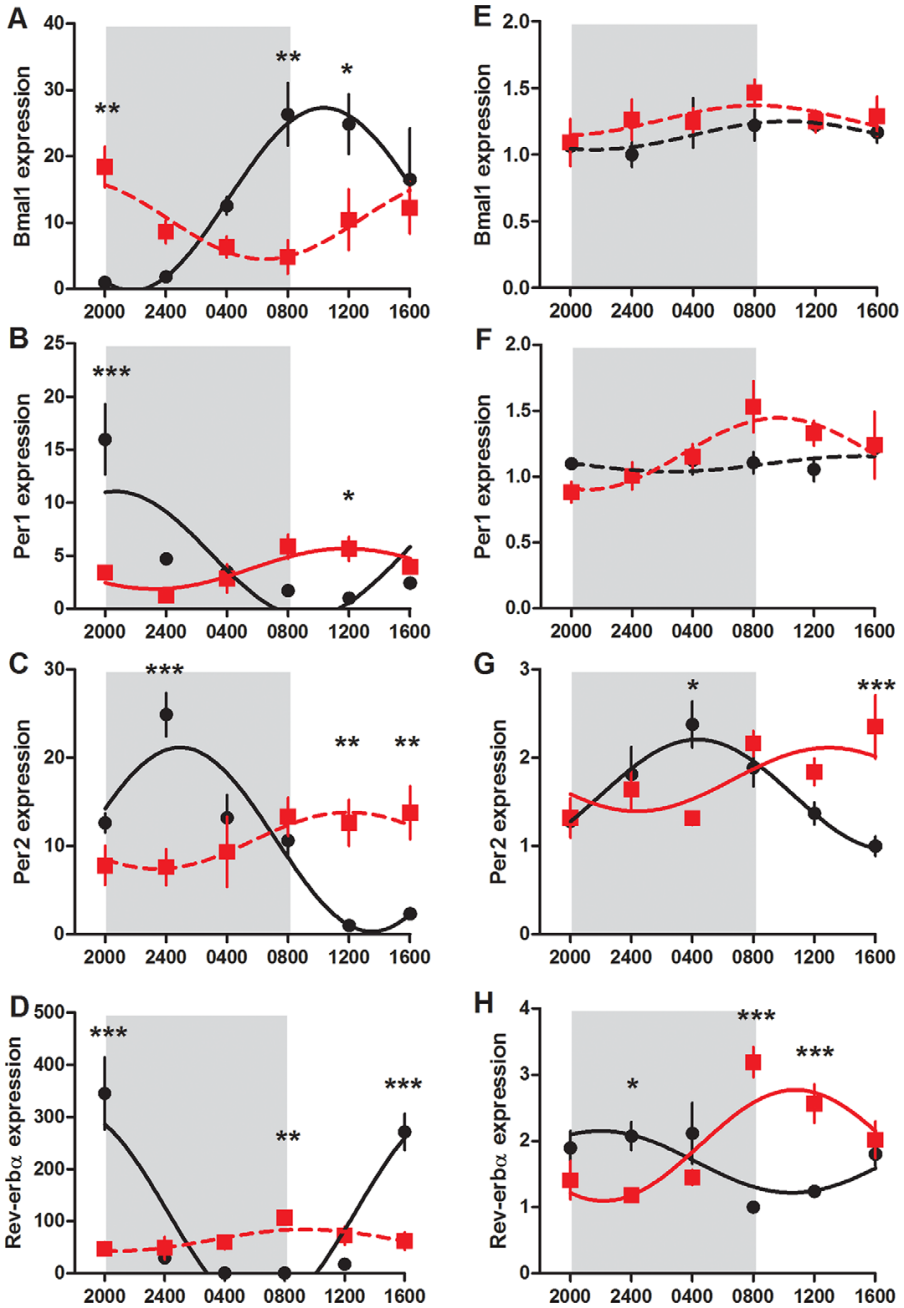
Clock gene expression in maternal and fetal liver. *Bmal1*, *Per1*, *Per2* and *Rev-erbα* mRNA expression in the maternal liver (a–d) and fetal liver (e–h) of control (black circle) and CPS (red square) dams at day 20 of gestation, 1 day after resumption of the normal photoperiod. Shading represents the time of darkness in both groups. Data has been fit to sine curve with the period constrained to 1, with solid lines representing a significant fit and dashed lines a non-significant fit. Data is mean ± SEM, n = 4–6, *P<0.05, **P<0.01, ***P<0.001.

The hepatic expression of *Bmal1* mRNA was rhythmic, changing 25 fold from a nadir at 2245 h to a peak 12 hours later at 1045 h (P<0.001, Figure 5). Conversely, the expression of *Bmal1* in the CPS treated animals was not rhythmic (P>0.05) despite changing from a peak at 1934 h to a nadir at 0724 h, in antiphase to that of the controls. The amplitude of this change was greatly reduced in the CPS dams, changing only 4 fold from peak to nadir. While ANOVA revealed no overall effect of treatment (P>0.05), *Bmal1* varied differently with time such that CPS dams had increased expression at 2000 h (P<0.01) and decreased expression at 0800 h (P<0.01) and 1200 h (P<0.05).

The expression of *Per1* mRNA in both the control and CPS dam livers was rhythmic (P<0.01), however, the amplitude and time of peak expression varied substantially between the two groups. In the control animals, peak *Per1* expression occurred at 2038 h, with an amplitude change of 16 fold. In the CPS dams however, *Per1* expression had peak levels at 0922 h, with an amplitude change from peak to trough of only 3 fold. Again there was no overall effect of treatment in the ANOVA (P>0.05), yet *Per1* varied differently with time such that CPS dams had reduced expression at 2000 h (P<0.001) and increased expression at 1200 h (P<0.05).

*Per2* expression was highly rhythmic and in antiphase to *Bmal1* in the control dams changing 19 fold from a trough at 1326 h to a peak at 0126 h (P<0.001). However, in the CPS dams, *Per2* expression was not significantly rhythmic (P>0.05), with minimal changes across the 24 hours. There was a significant treatment x time interaction (P<0.001) whereby CPS dams had reduced expression at 2400 h (P<0.001), and elevated levels at 1200 h and 1600 h (P<0.01).

*Rev-erbα* expression in the control dams changed rhythmically over the 24 hour period of sampling, from peak values at 1918 h, decreasing 350 fold to trough values at 0343 h (P<0.001). Alternatively, *Rev-erbα* expression in the CPS liver dams was not rhythmic (P>0.05), with amplitude changes from peak to trough being only 2 fold. ANOVA revealed a treatment effect (P<0.05), and a treatment x time interaction (P<0.001) whereby CPS dams had reduced expression at 2000 h and 1600 h (P<0.001) and increased expression at 0800 h (P<0.01).

### Maternal Liver Metabolic Gene Expression

**Figure 6:**
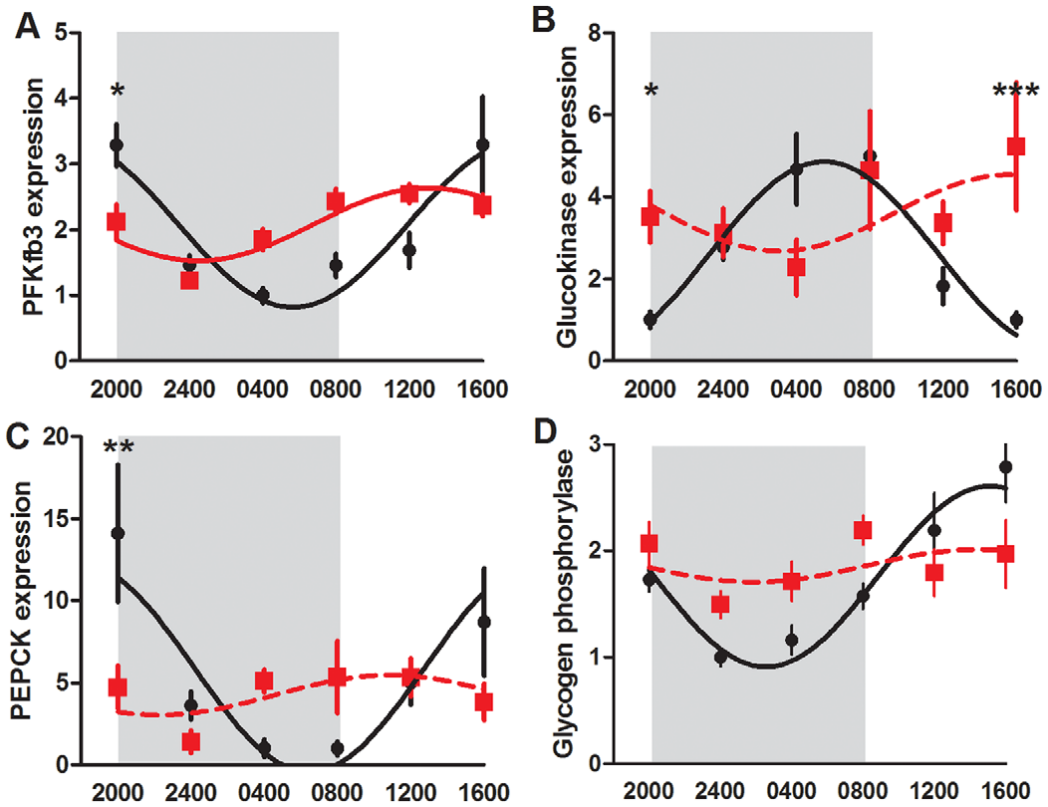
Circadian profile of metabolic gene expression in maternal liver. *PFKfb3* (a), *glucokinase* (b), *PEPCK* (c) and *glycogen phosphorylase* (d) mRNA expression in the maternal liver of control (black circle) and CPS (red square) dams at day 20 of gestation, 1 day after resumption of the normal photoperiod. Shading represents the time of darkness in both groups. Data has been fit to sine curve with the period constrained to 1, with solid lines representing a significant fit and dashed lines a non-significant fit. Data is mean ± SEM, n = 4–6 per treatment group per time point, *P<0.05, **P<0.01, ***P<0.001.

*6-phosphofructo-2-kinase/fructose-2,6-biphosphatase 3 (PFKfb3)* mRNA changed rhythmically over 24 hours, with data from both the control and CPS dams significantly fitting a sine curve (P<0.001, Figure 6). In the control animals, expression of this gene was elevated at the start of darkness, decreasing through the night, before rising slowly through the following day. In the CPS treated dams however, the phase of expression was advanced by 8.5 hours such that peak expression occurred just after the onset of the light period. ANOVA revealed a treatment x time interaction effect (P<0.001) whereby CPS dams had reduced expression at 2000 h (P<0.05). In contrast, *glucokinase*, *phosphoenolpyruvate carboxykinase (PEPCK)* and *glycogen phosphorylase* mRNA was only rhythmic in the control animals, becoming arrhythmic in the CPS dams. *Glucokinase* expression was significantly affected by treatment (P<0.05), with *post hoc* analyses revealing increased expression at 2000 h and 1600 h in the CPS dams (P<0.05 and P<0.001 respectively). There was no overall treatment effect on *PEPCK* mRNA, however expression varied differently with time (P<0.05) such that CPS dams had decreased expression at 2000 h (P<0.01). Similarly, *glycogen phosphorylase* was affected by treatment in a time dependent manner (P<0.01), however *post hoc* analyses with Bonferroni corrections do not reveal a significant difference at any individual time point (P>0.05).

### Fetal and Placental Gene Expression

**Figure 7:**
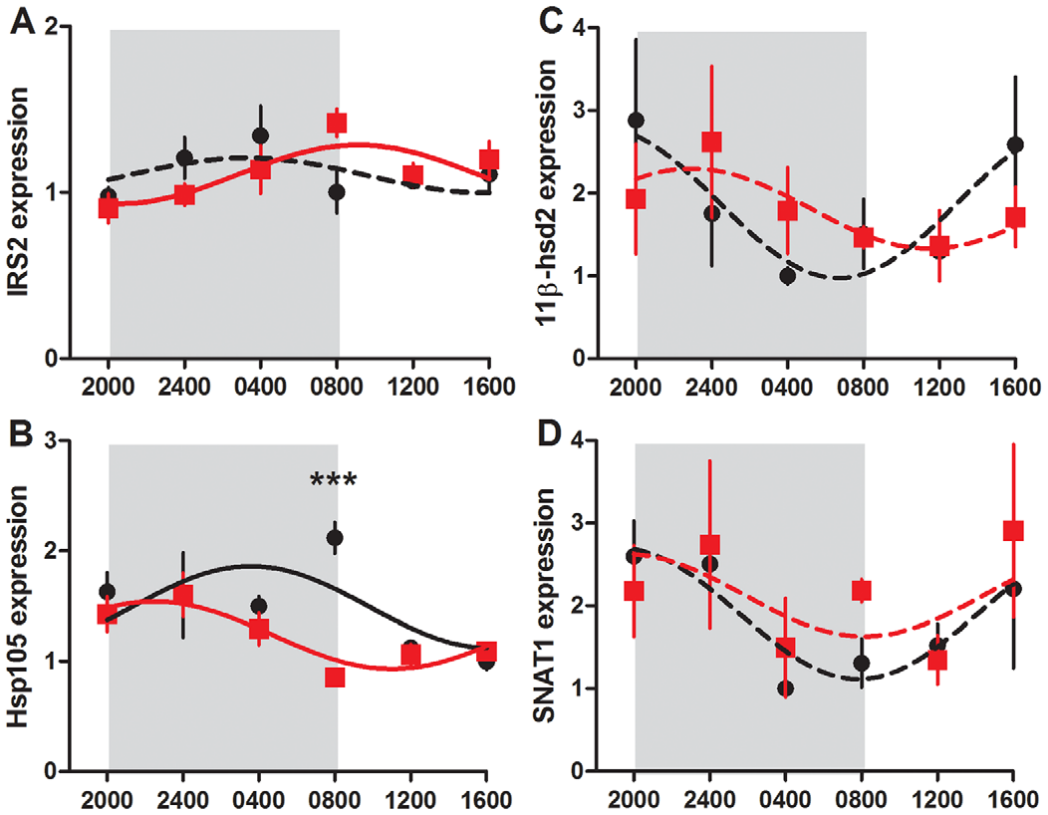
Fetal liver and placental labyrinth gene expression. *IRS2* (a), *Hsp105* (b) in fetal liver, and *11β-hsd2* (c), *SNAT1* (d) in placental labyrinth of control (black circle) and CPS (red square) dams at day 20 of gestation. Shading represents the time of darkness in both groups. Data has been fit to sine curve with the period constrained to 1, with solid lines representing a significant fit and dashed lines a non-significant fit. Data is mean ± SEM, n = 4–6 per treatment group per time point, ***P<0.001.

In the fetal liver, *Bmal1* and *Per1* mRNA was not rhythmically expressed, however in control and CPS animals, the expression of *Per2* and *Rev-erbα* significantly fitted a sine curve (P<0.05, Figure 5). Furthermore, the expression profiles of these genes in the control and CPS fetal livers were in antiphase. For the control animals, peak expression levels were observed during the time of darkness, whereas for CPS animals, peak levels occurred during the day. *Per2* mRNA expression varied differently with time (P<0.001) such that decreased expression was observed at 0400 h (P<0.05) and increased expression was observed at 1600 h (P<0.001) in the CPS fetal livers. For *Rev-erbα* mRNA, there was an overall treatment effect (P<0.05) with *post hoc* Bonferroni analyses revealing decreased expression at 2400 h (P<0.05) and increased expression at 0800 h and 1200 h (P<0.001) in the CPS fetal livers. *Insulin receptor substrate 2* (IRS2) mRNA in the fetal liver was affected by treatment in a time dependent manner (P<0.05, Figure 7), however *post hoc* Bonferroni analyses do not reach significance at any individual time point (P>0.05). *Hsp105* mRNA was affected by treatment (P<0.01), with CPS fetal livers showing reduced expression at 0800 h. Data from both control and CPS animals significantly fitted a sine curve (P<0.05).

The expression of placental *11β-hsd2* and *SNAT1* mRNA was examined in placental labyrinth (Figure 7). There was no effect of treatment on the expression of either of these genes (P>0.05). However, while neither *11β-hsd2* nor *SNAT1* expression in the CPS placentas significantly fitted a sine curve (P>0.05), the time dependent changes in the control placentas approached significance (p = 0.06 and 0.09 respectively).

## Discussion

We have previously shown that exposure of pregnant rats to chronic phase shifts of the lighting environment from conception through to early lactation negatively influences a range of metabolic parameters in the subsequent adult offspring [4]. In particular, at 3 months of age, male offspring had increased adiposity and hyperleptinaemia. By 12 months of age, the female offspring also displayed this phenotype, with the addition of hyperinsulinaemia and reduced glucose tolerance and insulin sensitivity. The aim of the current study was to gain an understanding of metabolic and other changes occurring in the pregnant dam throughout gestation caused by CPS exposure which may lead to the programming of metabolic disturbances in the fetus. We found that CPS exposure throughout gestation profoundly altered circadian rhythms of maternal corticosterone, glucose, insulin, leptin, free fatty acids, triglycerides and cholesterol as well as disrupting the timing of food intake and hepatic clock and metabolic gene expression. There was no effect on glucose tolerance, insulin sensitivity or total food consumption in the dams, and yet the rate of maternal weight gain was altered. Furthermore, CPS exposure *in utero* disrupted the timing of circadian and metabolic gene expression in the developing fetal liver.

The changes in weight gain in the CPS dams were unexpected, being reduced during the first week of gestation. However, as gestation progressed, the CPS dams went on to gain weight more rapidly, such that at the end of gestation they weighed only 4% less than the controls. It is conceivable that the reduction in weight gain observed during the early stages of gestation, a critical stage of embryo development, may have mediated the impact of this protocol on the metabolic health of the offspring. Experimental manipulation of weight gain through moderate caloric restriction during the first half of gestation has previously been shown to influence the metabolic health of rat offspring [6], [7]. Restricting calories to 20% of the control animals from conception to day 13 reduced initial body weight gains, but as gestation progressed and food access returned to *ad libitum*, the caloric restricted animals caught up so that by the end of gestation there was no significant difference. While this did not affect birth weight, the offspring went on to develop gender dependent hyperphagia, increased adiposity, hyperinsulinaemia and hyperleptinaemia by 5 months of age [6]. This phenotype was programmed through altered neuronal development of the hypothalamic circuits regulating feed intake [7]. It therefore appears that exposure to even moderate nutrient restriction during early gestation can have profound effects on the metabolic homeostasis of the adult offspring. In our study, the CPS protocol did not reduce total food intake at any stage of gestation, but rather changed the timing of food consumption, leading to reduced weight gain during the first week of gestation. This may have been a sufficient insult to program perturbed metabolic homeostasis in the offspring.

At day 20 of gestation, the CPS dams had significantly reduced retroperitoneal fat pad weights and a trend for decreased epigonadal fat weights. Furthermore, maternal liver weight was reduced at the start of the light period, in contrast to the control animals which had elevated liver weights at this time. The reduced fat pad and liver weight was not due to a reduction in these tissues in response to CPS exposure. Instead, the CPS dams failed to increase the size of these tissues in response to pregnancy at the same rate as the controls, as they still had greater fat and liver weights than that of the non-pregnant animals. The reduced liver weights observed at 1200 h may be due to increased hepatic glycogenolysis in response to low plasma glucose, insulin and leptin levels detected through the previous dark period. This is supported by the observation of increased *glycogen phosphorylase* mRNA in the preceding time points, a key enzyme involved in the release of glucose-1-phosphate from glycogen.

Exposure to the rapid changes in photoperiod has driven changes in the timing of food consumption leading to intermittent grazing rather than consolidated bouts of feeding during the dark period. With each photoperiod shift, the CPS dams are unable to adjust their pattern of food consumption to align with the new phase of darkness, leading to a disrupted profile of food consumption. Repeated exposure to these shifts may have led to decreased food efficiency as reflected in reduced weight gain and adipogenesis, particularly during the early stages of gestation. Weight gain during the later stages of gestation is a reflection of fetal and placental growth, as these tissues account for 25% of maternal weight at term [8]. Given that litter size, fetal weight, and placental weight were all unaffected by the CPS protocol, it is perhaps unsurprising that maternal weight normalised to the controls towards the end of gestation.

An analysis of the metabolic profile of the CPS and control dams at day 20 of gestation reveal profound changes to glucose, insulin, free fatty acids, triglycerides and cholesterol concentrations in plasma, consistent with disrupted timing of food consumption. In the control animals there were strong rhythms of these metabolites, with the exception of plasma triglycerides. However, in the CPS dams, the concentrations of these metabolites were either in antiphase or arrhythmic. Furthermore, the usual strong relationship between glucose and insulin as observed in the control animals was disrupted. Further analysis of the mRNA of some key hepatic enzymes involved in glycolysis and gluconeogenesis suggest a dampened profile of expression. While none of these enzymes were either up or down regulated, the observed arrhythmicity suggests major changes to these metabolic processes.

A rhythm in plasma leptin was observed in the control dams, with high levels at night during the time of feeding, and low levels during the day as previously described in non-pregnant rats [9]. The reduced levels of leptin detected in the CPS dams at night is likely due to the reduced adiposity of these animals, combined with altered timing of food consumption. The junctional zone of the placenta, however, also secretes leptin directly into the maternal blood [10], and therefore we cannot exclude the possibility that the reduced maternal leptin concentrations observed here are due to reduced placental secretion. Leptin plays an important role in fetal development, with its receptor active during neonatal life [11]. In particular, leptin regulates development of hypothalamic circuits regulating energy homeostasis in adult life [12]. Therefore, alterations to the secretion of this hormone from the dam may impact upon the developing offspring, leading to perturbed hypothalamic function. The importance of leptin in neuronal development can be demonstrated by examining *ob/ob* mice, with offspring showing reduced brain weight and altered projections and synaptic inputs to hypothalamic circuits that regulate feeding [13]. Furthermore, many of these outcomes can be rescued through early postnatal administration of leptin [13]. However, in our model overall maternal leptin levels were reduced by 20% rather than being completely absent. Additionally, unlike *ob/ob* mice, the offspring retain the ability to secrete their own leptin both *in utero* and postnatally. Nevertheless, the maternal hypoleptinaemia observed in our study may play a role in the programming of perturbed metabolic homeostasis in the offspring.

Rhythmic melatonin secretion is regulated through indirect neural projections from the suprachiasmatic nucleus (SCN) of the hypothalamus to the pineal [14], and therefore gives an indication of the phase of central SCN rhythmicity. We expected that exposure to rapid photoperiod changes would alter clock functions within the SCN and consequently melatonin secretion. However, melatonin maintained a normal profile of high secretion during darkness and low secretion during light. It has previously been shown that when animals are exposed to either phase delays or phase advances of the photoperiod, the SCN and consequently the timing of melatonin secretion gradually shifts over subsequent days so that eventually the internal clock is in phase with the external light environment [15]. Depending upon the direction and degree of the shift, this can take many days. Therefore, it was surprising to observe that melatonin secretion in the CPS dams had already aligned to the external photoperiod only 1 day after the previous photoperiod reversal. We hypothesise instead that the central SCN clock had never fully adjusted to the photoperiod changes before the lights had been reversed again, and therefore, loosely maintains time with the original photoperiod. Additionally, melatonin secretion of pregnant animals may be less responsive to photoperiod changes than non-pregnant animals. Only through regular sampling throughout the whole protocol will this question be answered. Interestingly, this profile of undisturbed melatonin secretion, despite changes to the timing of light exposure, activity and food consumption is also apparent in human shift workers [16], [17].

Nevertheless, we expect that at stages during the protocol, the melatonin secretion would be suppressed, particularly during the periods of 24 hours of light, as light exposure of sufficient intensity completely suppresses melatonin secretion [18], [19]. Therefore, we cannot rule out the possibility that reduced melatonin secretion plays some role in mediating the changes previously observed in the offspring. Indeed, a recent report has demonstrated the importance of maternal melatonin secretion plays in programming energy metabolism in the offspring [20]. Pups born to dams that were pinealectomised prior to gestation display a similar phenotype to that observed following CPS exposure, including glucose intolerance. Importantly, supplementation of the dams’ nightly drinking water with melatonin, and the concomitant elevation in nocturnal plasma melatonin, attenuated the negative effects of maternal pinealectomy on the offspring. Surprisingly, the negative consequences for the offspring appeared much earlier, with glucose intolerance becoming detectable at 4 months of age, compared to 12 months following the CPS protocol [4].

Compared to the aligned profiles of melatonin secretion in the control and CPS dams, there were large differences in corticosterone secretion between the two groups. Like melatonin, corticosterone secretion is regulated through efferent connections from the SCN. Neurons of the SCN terminate on cells of the PVN, leading to rhythmic CRH release, ACTH secretion from the pituitary and downstream rhythmic secretion of corticosterone from the adrenal (reviewed in [21]). Furthermore, the adrenal response to ACTH secretion also changes over 24 hours, mediated through both local clock mechanisms and SCN-mediated activation of the autonomic nervous system [21], [22]. Together this leads to a rhythmic profile of secretion with increased levels at the end of the light (sleep) period. However, unlike the pineal, the HPA axis is responsive to regulation at many levels via stress, food anticipation and negative feedback mechanisms [23], [24]. Therefore, it was not surprising to observe a disrupted profile of secretion in the CPS dams. The regulating signals driving arrhythmic corticosterone secretion were not identified in this study, but likely reflect altered sensitivity of the HPA axis to stimulation at all levels, as well as changes to the timing of incoming signals.

Regardless of the cause of the disrupted corticosterone secretion, the altered pattern can be expected to mediate important changes in maternal peripheral tissues and the developing fetus. Most peripheral tissues express glucocorticoid receptor (GR), which can then affect the transcription of many genes through both glucocorticoid response elements (GRE) or through interaction with other transcription factors [25]. PEPCK is an example of a gene possessing a GRE [26], the expression of which has been profoundly disrupted in response to CPS exposure. The sensitivity of peripheral tissues also fluctuates over 24 hours through changes in the level of receptor expression and GR-induced transcriptional activity [27], [28]. During development, appropriate glucocorticoid levels play a vital role in normal fetal development [29], with most fetal tissues expressing GR from early embryonic life [30]. The fetus is largely protected from excessive levels of glucocorticoids through the actions of placental 11β-hydroxysteroid dehydrogenase 2 (11β-hsd2), which converts glucocorticoids into their inert forms [31]. Nevertheless, disproportionate levels of glucocorticoids during both early and late fetal development can have profound effects on the programming of adult metabolism [32], [33]. Usually these effects are mediated through reduced growth and subsequent smaller birth weights, although this is not always the case, particularly when exposure occurs during early gestation [34]. While we didn’t observe elevated overall levels of corticosterone in maternal blood, reduced *11β-hsd2* mRNA in placenta, or reduced growth rates of the fetus, it is possible that the disrupted timing of secretion may in some way negatively impact key developmental processes.

Given that the CPS protocol manipulates the dominant zeitgeber to the circadian system (ie light), it was of interest to investigate the impact of this protocol on clock genes in both maternal and fetal tissues. Constant light exposure to pregnant dams has previously been shown to dampen the expression of not only melatonin secretion, but also maternal and fetal clock gene expression, which can be regained through nocturnal melatonin administration [35]. As expected, we found that in the control dams, the expression of *Bmal1*, *Per1, Per2* and *Rev-erbα* mRNA in the maternal liver was rhythmic, with high amplitude changes in expression from peak to trough. Importantly, the pattern of expression was such that *Bmal1* mRNA was in antiphase to *Per2*, consistent with a functional transcription-translation feedback loop existing in this tissue. When exposed to the CPS protocol however, pregnant dams experienced significant disruption to clock gene expression; the expression of *Bmal1*, *Per2*, and *Rev-erbα* did not significantly fit a sine curve, and the amplitude changes were greatly reduced. Alternatively, within the fetal liver of control animals, *Per2* and *Rev-erbα* mRNA expression was rhythmic with small amplitude changes from peak to trough, but not *Bmal1* mRNA expression. Given the importance of *Bmal1* in driving rhythmic clock gene expression, and the necessity of this gene in maintaining activity of a transcription-translation feedback loop, it was surprising to then observe robust rhythms of *Per2* and *Rev-erbα* mRNA. Interestingly, CPS fetal livers also rhythmically expressed *Rev-erbα* and *Per2* mRNA, in antiphase to the controls.

What could be driving the rhythmic transcription of these genes, if not the core circadian feedback loop? Schibler and colleagues developed mice with a conditionally active, hepatocyte-specific *Rev-erbα* transgene, which led to suppression of the core circadian transcriptional feedback loop through suppression of *Bmal1* within the liver but not the rest of the body [36]. Under these conditions, circadian expression of the core clock genes was arrhythmic, with the notable exception of *Per2*, which maintained an identical profile of expression to that of the controls. This suggests that rather than being solely reliant on a functional transcription-translation feedback loop to maintain rhythmicity, *Per2* is instead responding to systemic cues. In the context of the fetal liver developing within a rhythmic maternal system, the ability of *Per2* to sense or detect maternal rhythms allows fetal tissue to maintain rhythmicity in the absence of an endogenous circadian transcriptional feedback loop. The molecular mechanisms, however, that transmit this information are as yet unknown, but candidates include core body temperature through heat shock factors, maternal and/or fetal glucocorticoids, maternal melatonin and substrate concentrations. Unfortunately, fetal corticosterone concentrations were not assessed in this study, but may have shed some light on this question, particularly given recent demonstrations of the importance of fetal glucocorticoids in conveying maternal rhythmicity to the fetus [35]. Interestingly, we found that the expression of the heat shock protein *Hsp105* also changed rhythmically in the control and CPS fetal livers, but with a different phase of expression. This suggests that heat shock factors may mediate the rhythmicity of *Per2* and *Rev-erbα* in the fetus, particularly given the presence of heat shock response elements in the promoter of at least *Per2*. It cannot be dismissed that disruptions to the timing of circadian gene expression within the developing fetus, as observed here in the fetal liver, may play some role in the programming of adult metabolic disease.

### Conclusions

We have shown that exposure of pregnant dams to chronic phase shifts of the photoperiod profoundly alters the normal circadian pattern of food consumption, endocrine secretion and metabolite concentrations, as well as metabolic and circadian gene expression in the mother and fetus. Together these disruptions are likely to have a negative effect on the developing fetus. However, the reduced weight gain of the dams during a key stage of early embryo development is likely to play a major role in the previously observed offspring phenotype of increased adiposity, hyperinsulinaemia, hyperleptinaemia and poor glucose tolerance. Intriguingly, the reduced maternal weight gain occurred independently of total food consumption, suggesting that merely changing the timing of feeding and the downstream metabolic processes required to utilise those nutrients, can lead to an altered metabolic phenotype in the adult offspring. In the context of human pregnancies, maternal shift work also severely impacts upon the timing of activity and food consumption. If the effects seen here in a rat model translate into the human situation, then merely disrupting the timing of food consumption may have lasting impacts upon the developing baby, independent of birth weight, which may predispose those offspring to the development of metabolic disorders as adults.

## Supporting Information

Figure S1 **Schematic of the control and CPS protocols.** Upon the presence of sperm in a vaginal smear, female rats were exposed to control lighting conditions (12l:12D, lights on at 0800 h, left panel), or the CPS protocol whereby the photoperiod was reversed twice every week (right panel). Food and water was provided *ad libitum* throughout the protocol. Blue line, positive vaginal smear; green lines, time of tissue collections for hormonal, metabolite and gene expression analyses; red block, time of glucose and insulin tolerance tests.
(TIF)

Table S1 **Primer sequences.**
(DOCX)

Table S2 **Cumulative food consumption as measured by the LabMaster system.**
(DOCX)
