# Supplementary figures and images for: Characterisation of the Maternal Response to Chronic Phase Shifts during Gestation in the Rat: Implications for Fetal Metabolic Programming
Source: PLoS One. 2013 Jan 14;8(1):e53800. doi: 10.1371/journal.pone.0053800 (PMC3544759; doi:10.1371/journal.pone.0053800)

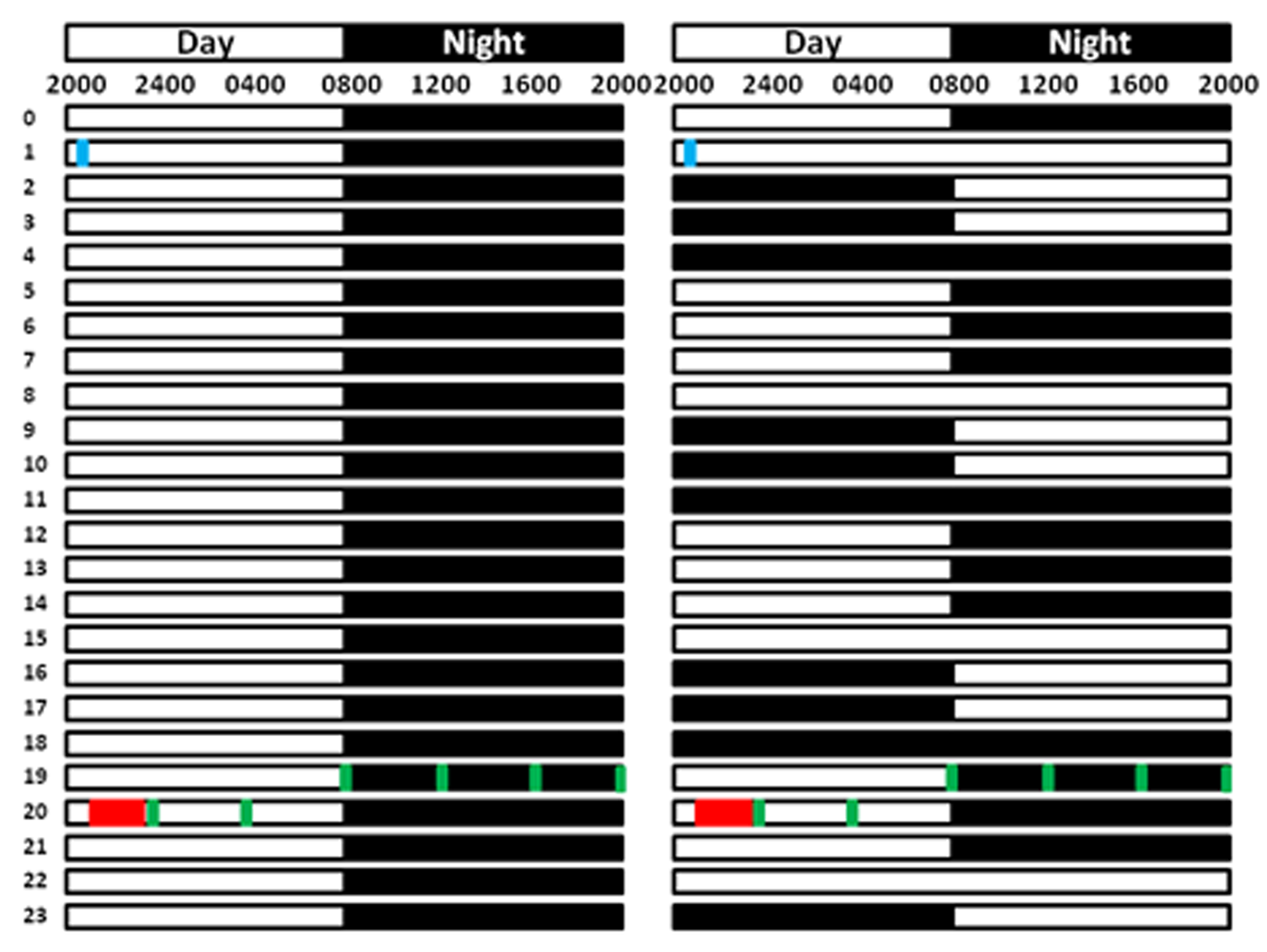

Supplement: Figure S1 — Schematic of the control and CPS protocols. Upon the presence of sperm in a vaginal smear, female rats were exposed to control lighting conditions (12l:12D, lights on at 0800 h, left panel), or the CPS protocol whereby the photoperiod was reversed twice every week (right panel). Food and water was provided ad libitum throughout the protocol. Blue line, positive vaginal smear; green lines, time of tissue collections for hormonal, metabolite and gene expression analyses; red block, time of glucose and insulin tolerance tests. (TIF) [file pone.0053800.s001.tif]
