# Supplementary material for: Characterisation of the Maternal Response to Chronic Phase Shifts during Gestation in the Rat: Implications for Fetal Metabolic Programming
Source: PLoS One. 2013 Jan 14;8(1):e53800. doi: 10.1371/journal.pone.0053800 (PMC3544759; doi:10.1371/journal.pone.0053800)
Supplement: Table S1 — Primer sequences. (DOCX) [file pone.0053800.s002.docx]

Supplementary Figure 1 Primer sequences

| **Gene** | **Accession Number** | **Primer** | **Sequence 5’ to 3’** |
| --- | --- | --- | --- |
| **r*ßactin*** | NM­_031144 | Forward  Reverse | cctctgaaccctaaggccaa  agcctggatggctacgtaca |
| **r*Per1*** | NM_001034125 | Forward  Reverse | TGGTAAAGCACCAGGGACAAC  GAAGGACTTTGGCCTTGAATGTAC |
| **r*Per2*** | NM_031678 | Forward  Reverse | AGCAGTCCCCTACAGCTTAACCT  CCGAGATGCGCCAGATGT |
| **r*Bmal1*** | NM_024362 | Forward  Reverse | TCCACAGCACAGGTACTTGAA  TTGCAACGAGGCAGCTCAGAT |
| **r*Rev-erbα*** | NM_001113422 | Forward  Reverse | acagctgacaccacccagatc  catgggcataggtgaagatttct |
| **r*PFKfb3*** | NM_057135 | Forward  Reverse | AGGCAAGAAGTTCGCCAATG  GCGGTCTGGATGGTACTTTTCA |
| **r*Glucokinase*** | NM_012565 | Forward  Reverse | CTCTGTCACCGACTGCGACAT  TGCGATTTATGACCCCAGCTA |
| **r*PEPCK*** | NM_198780 | Forward  Reverse | CCCTTAAAAAAGCCTTTGGTCAA  CCGCTTCCGAAGGAGATGA |
| **r*Glycogen phosphorylase*** | NM_022268 | Forward  Reverse | AGTCAGTCAGCTGTATATGAATCAA  AGATCGGAAGGCTCCATGTT |
| ***R11β-hsd2*** | NM_017081 | Forward  Reverse | TTTTGGCAAGGAGACAGCTAAGA  ATCGGGCACGCAGTTCTAGA |
| ***rSNAT1*** | NM_138832 | Forward  Reverse | GAAGTGGAGAACGGCCAGATAA  CCTGGAATATACTCGTCGCATTT |
| ***rHsp105*** | NM_001011901 | Forward  Reverse | GTGTCTGCCTGTGCTTTTAACAA  TGCTCTACGAGCTTCTCGTCAA |
| ***rIRS2*** | NM_001168633 | Forward  Reverse | CAAGAACCTGACCGGTGTATACC  GCCCACCTCGATGAAGAAGA |
