# Supplementary material for: Characterisation of the Maternal Response to Chronic Phase Shifts during Gestation in the Rat: Implications for Fetal Metabolic Programming
Source: PLoS One. 2013 Jan 14;8(1):e53800. doi: 10.1371/journal.pone.0053800 (PMC3544759; doi:10.1371/journal.pone.0053800)
Supplement: Table S2 — Cumulative food consumption as measured by the LabMaster system. (DOCX) [file pone.0053800.s003.docx]

Table S2 Cumulative food consumption as measured by the LabMaster system

|  | Day 8 | Day 15 | Day 22 |
| --- | --- | --- | --- |
| Control | 117 ± 4 | 249 ± 9 | 407 ± 13 |
| CPS | 127 ±11 | 254 ± 54 | 414 ± 44 |
| Data are mean ± SEM, control n=4, CPS n=5. | | | |
